# Supplementary material for: Effect of stimulus orientation and intensity on short-interval intracortical inhibition (SICI) and facilitation (SICF): A multi-channel transcranial magnetic stimulation study
Source: PLoS One. 2021 Sep 22;16(9):e0257554. doi: 10.1371/journal.pone.0257554 (PMC8457500; doi:10.1371/journal.pone.0257554)
Supplement: S4 Table — The results are presented as follows, for a fixed conditioning stimulus orientation (CSO), test stimulus orientation (TSO), and interstimulus interval (ISI), we compute the motor evoked potential (MEP) amplitude ratio (MEP ratio) between the tested CSI. Each comparison has a standard error (SE), degrees of freedom (DoF), t–ratio, and p-value. Tested stimulus orientations were anteromedial (AM) and posteromedial (PM); CSI is given as a percentage of the orientation-specific resting motor threshold (RMT). p-values in bold are smaller than the threshold for statistical significance (0.05). (DOCX) [file pone.0257554.s004.docx]

**Supplementary Table 4.** Multiple comparisons between condition stimulus intensities (CSI). The results are presented as follows, for a fixed conditioning stimulus orientation (CSO), test stimulus orientation (TSO), and interstimulus interval (ISI), we compute the motor evoked potential (MEP) amplitude ratio (MEP ratio) between the tested CSI. Each comparison has a standard error (SE), degrees of freedom (DoF), *t*–ratio, and *p*-value. Tested stimulus orientations were anteromedial (AM) and posteromedial (PM); CSI is given as a percentage of the orientation-specific resting motor threshold (RMT). *p*-values in bold are smaller than the threshold for statistical significance (0.05).

| CSO | TSO | ISI (ms) | Comparison  CSI_1_ / CSI_2_ (% RMT) | MEP ratio (CSI_1_ / CSI_2_) | SE | DoF | *t*-ratio | *p*-value |
| --- | --- | --- | --- | --- | --- | --- | --- | --- |
| AM | PM | 1.5 | 50 / 70 | 1.58 | 0.50 | 10.58 | 1.47 | 0.23 |
| AM | PM | 1.5 | 50 / 90 | 0.30 | 0.11 | 9.52 | –3.38 | **0.01** |
| AM | PM | 1.5 | 50 / 110 | 0.09 | 0.03 | 10.98 | –7.95 | **< 0.001** |
| AM | PM | 1.5 | 70 / 90 | 0.19 | 0.04 | 15.65 | –7.11 | **< 0.001** |
| AM | PM | 1.5 | 70 / 110 | 0.06 | 0.01 | 17.09 | –12.67 | **< 0.001** |
| AM | PM | 1.5 | 90 / 110 | 0.31 | 0.09 | 11.29 | –4.04 | **< 0.001** |
| AM | PM | 2.7 | 50 / 70 | 7.00 | 2.19 | 10.58 | 6.22 | **< 0.001** |
| AM | PM | 2.7 | 50 / 90 | 2.23 | 0.80 | 9.52 | 2.24 | 0.08 |
| AM | PM | 2.7 | 50 / 110 | 0.16 | 0.05 | 10.98 | –6.08 | **< 0.001** |
| AM | PM | 2.7 | 70 / 90 | 0.32 | 0.07 | 15.65 | –4.87 | **< 0.001** |
| AM | PM | 2.7 | 70 / 110 | 0.02 | 0.01 | 17.09 | –16.76 | **< 0.001** |
| AM | PM | 2.7 | 90 / 110 | 0.07 | 0.02 | 11.29 | –8.98 | **< 0.001** |
| PM | PM | 1.5 | 50 / 70 | 1.92 | 0.60 | 10.58 | 2.09 | 0.10 |
| PM | PM | 1.5 | 50 / 90 | 0.54 | 0.19 | 9.53 | –1.74 | 0.17 |
| PM | PM | 1.5 | 50 / 110 | 0.17 | 0.05 | 10.98 | –5.93 | **< 0.001** |
| PM | PM | 1.5 | 70 / 90 | 0.28 | 0.07 | 15.68 | –5.43 | **< 0.001** |
| PM | PM | 1.5 | 70 / 110 | 0.09 | 0.02 | 17.09 | –10.83 | **< 0.001** |
| PM | PM | 1.5 | 90 / 110 | 0.31 | 0.09 | 11.30 | –3.97 | **< 0.001** |
| PM | PM | 2.7 | 50 / 70 | 11.01 | 3.45 | 10.58 | 7.67 | **< 0.001** |
| PM | PM | 2.7 | 50 / 90 | 1.86 | 0.67 | 9.53 | 1.74 | 0.17 |
| PM | PM | 2.7 | 50 / 110 | 0.17 | 0.05 | 10.98 | –5.90 | **< 0.001** |
| PM | PM | 2.7 | 70 / 90 | 0.17 | 0.04 | 15.68 | –7.56 | **< 0.001** |
| PM | PM | 2.7 | 70 / 110 | 0.02 | 0.00 | 17.09 | –18.53 | **< 0.001** |
| PM | PM | 2.7 | 90 / 110 | 0.09 | 0.03 | 11.30 | –8.17 | **< 0.001** |
| AM | AM | 1.5 | 50 / 70 | 0.86 | 0.27 | 10.58 | –0.47 | 0.69 |
| AM | AM | 1.5 | 50 / 90 | 0.21 | 0.07 | 9.52 | –4.42 | **< 0.001** |
| AM | AM | 1.5 | 50 / 110 | 0.09 | 0.03 | 10.98 | –8.04 | **< 0.001** |
| AM | AM | 1.5 | 70 / 90 | 0.24 | 0.06 | 15.65 | –6.11 | **< 0.001** |
| AM | AM | 1.5 | 70 / 110 | 0.10 | 0.02 | 17.09 | –10.09 | **< 0.001** |
| AM | AM | 1.5 | 90 / 110 | 0.43 | 0.13 | 11.29 | –2.86 | **0.03** |
| AM | AM | 2.7 | 50 / 70 | 3.82 | 1.19 | 10.58 | 4.28 | **< 0.001** |
| AM | AM | 2.7 | 50 / 90 | 1.23 | 0.44 | 9.52 | 0.59 | 0.65 |
| AM | AM | 2.7 | 50 / 110 | 0.19 | 0.06 | 10.98 | –5.47 | **< 0.001** |
| AM | AM | 2.7 | 70 / 90 | 0.32 | 0.08 | 15.65 | –4.81 | **< 0.001** |
| AM | AM | 2.7 | 70 / 110 | 0.05 | 0.01 | 17.09 | –13.26 | **< 0.001** |
| AM | AM | 2.7 | 90 / 110 | 0.16 | 0.05 | 11.29 | –6.33 | **< 0.001** |
| PM | AM | 1.5 | 50 / 70 | 1.44 | 0.45 | 10.61 | 1.16 | 0.36 |
| PM | AM | 1.5 | 50 / 90 | 0.48 | 0.17 | 9.54 | –2.06 | 0.11 |
| PM | AM | 1.5 | 50 / 110 | 0.17 | 0.05 | 11.00 | –5.81 | **< 0.001** |
| PM | AM | 1.5 | 70 / 90 | 0.33 | 0.08 | 15.72 | –4.69 | **< 0.001** |
| PM | AM | 1.5 | 70 / 110 | 0.12 | 0.03 | 17.13 | –9.38 | **< 0.001** |
| PM | AM | 1.5 | 90 / 110 | 0.36 | 0.11 | 11.30 | –3.45 | **0.01** |
| PM | AM | 2.7 | 50 / 70 | 6.50 | 2.03 | 10.58 | 5.98 | **< 0.001** |
| PM | AM | 2.7 | 50 / 90 | 1.83 | 0.66 | 9.52 | 1.69 | 0.18 |
| PM | AM | 2.7 | 50 / 110 | 0.16 | 0.05 | 10.98 | –6.14 | **< 0.001** |
| PM | AM | 2.7 | 70 / 90 | 0.28 | 0.07 | 15.65 | –5.39 | **< 0.001** |
| PM | AM | 2.7 | 70 / 110 | 0.02 | 0.01 | 17.09 | –16.51 | **< 0.001** |
| PM | AM | 2.7 | 90 / 110 | 0.09 | 0.03 | 11.29 | –8.37 | **< 0.001** |
